# Prediction of a common structural scaffold for proteasome lid, COP9-signalosome and eIF3 complexes

**DOI:** 10.1186/1471-2105-6-71

**Published:** 2005-03-24

**Authors:** Hartmut Scheel, Kay Hofmann

**Affiliations:** 1 Bioinformatics Group, Memorec Biotec GmbH, Stöckheimer Weg 1, D-50829 Köln, Germany

## Abstract

**Background:**

The 'lid' subcomplex of the 26S proteasome and the COP9 signalosome (CSN complex) share a common architecture consisting of six subunits harbouring a so-called PCI domain (proteasome, CSN, eIF3) at their C-terminus, plus two subunits containing MPN domains (Mpr1/Pad1 N-terminal). The translation initiation complex eIF3 also contains PCI- and MPN-domain proteins, but seems to deviate from the 6+2 stoichiometry. Initially, the PCI domain was defined as the region of detectable sequence similarity between the components mentioned above.

**Results:**

During an exhaustive bioinformatical analysis of proteasome components, we detected multiple instances of tetratrico-peptide repeats (TPR) in the N-terminal region of most PCI proteins, suggesting that their homology is not restricted to the PCI domain. We also detected a previously unrecognized PCI domain in the eIF3 component eIF3k, a protein whose 3D-structure has been determined recently. By using profile-guided alignment techniques, we show that the structural elements found in eIF3k are most likely conserved in all PCI proteins, resulting in a structural model for the canonical PCI domain.

**Conclusion:**

Our model predicts that the homology domain PCI is not a true domain in the structural sense but rather consists of two subdomains: a C-terminal 'winged helix' domain with a key role in PCI:PCI interaction, preceded by a helical repeat region. The TPR-like repeats detected in the N-terminal region of PCI proteins most likely form an uninterrupted extension of the repeats found within the PCI domain boundaries. This model allows an interpretation of several puzzling experimental results.

## Background

In eukaryotic organisms, there exist at least three distinct multi-protein assemblies that are jointly referred to as 'PCI complexes' [1] and have a similar subunit architecture despite their fundamentally different function: i) the proteasome lid, a subcomplex of the 19S proteasome regulator and the 26S proteasome, ii) the COP9 signalosome or CSN complex, and iii) the eukaryotic translation initiation factor eIF3. As a common feature, these complexes are composed of multiple subunits harbouring the PCI domain, named after the three participating complexes [2], sometimes also referred to as the PINT domain [3]. Other subunits of these complexes are characterized by a second shared homology domain called MPN (Mpr1-Pad1 N-terminal) [2,3].

Among these complexes, the proteasome lid and the CSN share a particular degree of analogy. Both complexes consist of eight core subunits, six of them of the PCI class and two of the MPN class. As described previously [1] and summarized in table 1, there is a clear 1:1 correspondence between the paralogous sets of PCI and MPN subunits. In addition, a similar ring-shaped structure was observed for the two complexes [4] and there is evidence that in those rings paralogous subunits occupy equivalent positions [5]. By contrast, the eIF3 complex has a smaller number of PCI subunits (table 1) and its two MPN subunits are absent in several unicellular eukaryotes. Unlike the proteasome lid and the CSN, the eIF3 complex contains a number of non-PCI/non-MPN subunits, which are required for its function in translation.

Despite the common homology domains and a similar structure, the functions of the three PCI complexes are very different. The proteasome lid, in combination with the 'base' complex containing a hexameric ring of AAA-ATPases, forms the 19S regulatory particle, which in turn constitutes an essential subcomplex of the 26S proteasome [6]. The lid complex contains an intrinsic deubiquitinating activity, which is encoded by the MPN subunit Rpn11 that has the hallmarks of a metalloprotease [7-9]. No specific function has been described for the PCI subunits of the lid. The CSN complex has been first described as a regulator of photomorphogenesis in plants, but seems to regulate diverse cellular processes like signal transduction, regulation of transcription or cell proliferation [10,11]. Csn5, an MPN-bearing subunit of the signalosome, which is analogous to Rpn11, also encodes a metalloprotease that is essential for the removal of the ubiquitin-like protein Nedd8 from cullins [12] The third PCI complex, the translation initiation factor eIF3, promotes the formation of preinitiation complexes and works as a scaffold by binding to other initiation factors, to ribosomes and to mRNA [13,14]. Both MPN subunits of eIF3 lack the residues necessary for metal binding [8,15] and are most likely catalytically inactive.

So far, the metal-containing MPN subunits and the non-PCI/non-MPN portion of the complex constitute the only known carriers of functionality. The PCI proteins themselves seem to be the main building blocks of the complexes, a fact already suggested by their high abundance. There are several hints that the PCI subunits are crucial for proper complex assembly [16-19]. The MPN subunits of the three complexes are rather well conserved and the detection of MPN domains and their boundaries is relatively straightforward. By contrast, the degree of conservation between PCI subunits is highly variable. Sequence similarity between the corresponding subunits of proteasome lid and CSN is generally easy to spot, while the detection of similarity between other paralogous PCI subunits typically requires sophisticated sequence comparison approaches, such as the generalized profile method [2,20]. A particular challenge is the detection of the highly divergent PCI domains in the budding yeast CSN-like complex [21] and those of the eIF3 complex, where only three PCI subunits could be detected in the initial survey [2]. Due to this difficulties, it is to be expected that there are still a number of highly divergent PCI domain proteins in eukaryotic genomes, which have eluded detection so far. A second issue in the bioinformatical definition of the PCI domain concerns the position of its N-terminal boundary. In general, homology domains are thought to correspond to structural domains in the sense of autonomous folding units; they are typically characterized by a pronounced loss of sequence similarity at the domain boundaries. While this is true for the PCI domain C-terminus, the N-terminal domain boundary is blurred through a gradual decay in sequence similarity instead of a sharp drop. As a consequence, different PCI domain boundaries have been used in the literature [2,3] and in various domain databases like PROSITE [22], Pfam [23] and SMART [24]. The corresponding accession numbers are PS50250, PF01399 and SM00088, respectively.

During an exhaustive bioinformatical analysis of proteasome subunits and other components of the ubiquitin/proteasome system, we obtained two independent results jointly suggesting that a structure-based redefinition of the PCI domain is appropriate: on one hand, we detected multiple instances of TPR-like repeats in the N-terminus of many PCI proteins, which suggests that the homology between the proteasome and CSN components is not restricted to the PCI domain itself. On the other hand, we detected a previously overlooked PCI domain in the novel eIF3 subunit eIF3k [25]. Most interestingly, an X-ray structure of eIF3k has been published recently [26]. Based on this structure and on our alignment data, we suggest a bipartite consensus model for the canonical PCI proteins, consisting of a C-terminal 'winged helix' domain preceded by an extended helical repeat region. We use this model to re-evaluate some bioinformatical and experimental findings that have been enigmatic so far.

## Results

### TPR-like helical repeats in PCI proteins

**Figure 1:**
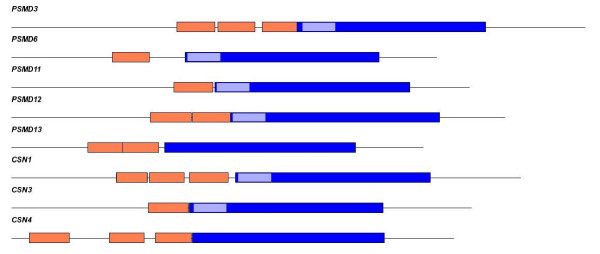
**TPR-like motifs upstream and inside the PCI domain**. The proposed domain topologies of selected human PCI proteins were investigated by profile techniques. Besides the common PCI domain (blue) short stretches of ~35 aa each are depicted in orange and light blue. These stretches show weak to medium similarity to TPR segments in established TPR proteins and merge seamlessly into the PCI domain in several PCI subunits. Corresponding accession numbers are listed in figure 2.

In most PCI proteins, the canonical PCI domain occupies a region of approximately 190 residues close to the carboxy-terminus of the sequence. The N-terminal non-PCI portion of the proteins is moderately conserved between species and only poorly conserved between different PCI subunits – even between the analogous subunits of the lid and the CSN. Upon submitting those PCI proteins to profile- or HMM-based domain detection services, no significant matches were obtained for the N-termini of the proteins. However, the PROSITE [27] profile for the tetratrico-peptide repeat (TPR) yielded a number of closely sub-significant matches in multiple PCI proteins, e.g. Rpn7 from *S. bayanus *(P value = 0.01, Ref [28]) and Csn1 from *E. histolytica *(P value = 0.06, Uniprot: Q8WQ58). The TPR repeat family [29] is very heterogeneous, and TPR motif descriptors such as the PROSITE profile are known to miss several instances of bona fide TPR repeats. Upon closer inspection, most PCI proteins exhibit multiple regions of similarity to profiles derived from established TPR repeats (matches schematically shown in figure 1), although the similarity scores for each of the single regions do not reach statistical significance. No relevant similarity scores were obtained for other helical repeat motifs, such as HEAT or Armadillo repeats.

To further investigate if a TPR-like structure should be assumed for the N-terminal portions of all PCI proteins, we performed a secondary structure prediction for each of the protein families individually. To that aim, we constructed multiple alignments for representative members of each subunit family and submitted the alignment to PHD and JPred prediction servers [30,31]. As a result, all PCI subunits of lid and CSN are predicted to adopt an all-helical secondary structure upstream of the PCI domain. Interestingly, these helical regions merge seamlessly into the PCI domain, at least if the longer PCI versions of PROSITE and Pfam are used. This finding is in agreement with the observation of several regions with weak TPR-similarity within the N-terminal part of the PCI domain itself (see figure 1). Further support for a TPR-like structure comes from a sequence-based fold recognition for lid and CSN subunits using the Superfamily-service [32]. Several subunits like Rpn7 from budding yeast and human Csn1 were found to have significant scores for the TPR fold upstream of the PCI domain (data not shown).

The predicted all-helical secondary structure of the non-PCI portion of lid and CSN subunits consists of several short helices that appear to occur in pairs. To test whether those bi-helical segments correspond to the structural elements of a TPR-like repeat, we selected several examples starting immediately upstream of the predicted PCI domains. When multiple alignments of those bi-helical segments were used for profile construction and in subsequent database searches several bona fide TPR proteins were found to match within the TPR region, with the bi-helices being in the correct TPR register, these segments were also classified as TPR-like. No matches to established HEAT- or Armadillo-repeat proteins were found, demonstrating that the scores are not just caused by an arbitrary helical repeat arrangement.

It should be pointed out that none of the singular observations described above is able to prove a statistically significant sequence relationship between the N-terminal portions of PCI proteins and true TPR-repeats. Taken as a whole, the results strongly suggest that there is a general tendency of PCI domains to be preceded by an α-helical repeat structure that has at least some specific relationship to the tetratrico-peptide repeat.

### A previously unrecognized PCI domain in eIF3k

In the first surveys of recognizable PCI domains, only three PCI subunits of the eIF3 complex had been detected [2]. More recently, a number of novel eIF3 components have been identified: eIF3j [33], eIF3k [25] and eIF3l [34]. Among these novel subunits, only eIF3l has been reported to harbour a PCI domain [34], interestingly also preceded by a TPR-region. In order to find further indications of divergent PCI domains, we performed a thorough profile analysis of all uncharacterized eIF3 subunits.

**Figure 2:**
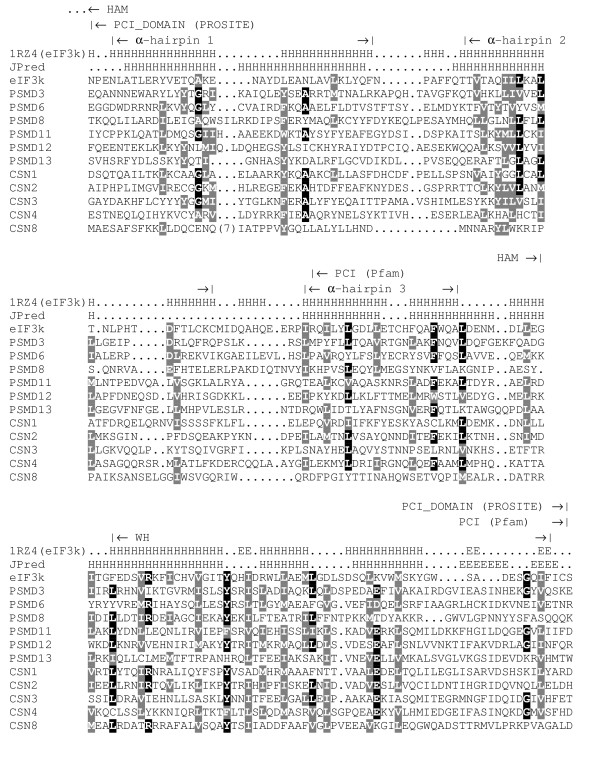
Multiple sequence alignment of human PCI subunits from proteasome lid, CSN and eIF3. Shown are only the segments matched by the PROSITE PCI domain. Conserved residues printed on black background were found in at last 50 % of ~60 PCI proteins from selected species, from which only human representatives are shown. Grey background was assigned to positions occupied by residues with similar physicochemical properties in at least 50 % of the sequences. The alignment was shaded using BOXSHADE . Above the PCI alignment secondary structure prediction as calculated from JPred [31] is presented. In these calculations sequences of eIF3k homologues were not included. Secondary structure elements of eIF3k as derived from PDB structure 1RZ4 are shown in a separate row. The abbreviations denote the following secondary structure types: E extended (sheet) and H helix. In addition, structural subdomain classification ('HAM', 'WH') as described in Wei et al. [26] and domain boundaries according to PCI profiles from PROSITE and Pfam are provided. Sequence names correspond to the following SwissProt database entries: eIF3k (Q9UBQ5), PSMD3 (O43242), PSMD6 (Q15008), PSMD8 (P48556), PSMD11 (O00231), PSMD12 (O00232), PSMD13 (Q9UNM6), CSN1 (Q13098), CSN2 (P61201), CSN3 (Q9UNS2), CSN4 (Q9BT78), CSN8 (Q99627).

A generalized profile was constructed from the conserved portion of representative eIF3k orthologs from vertebrates, invertebrates, plants and fungi. After a scaling step, the resulting profile was run against a nonredundant protein database. Apart from the eIF3k proteins already used for profile construction, the only other sequences matching with significance were selected PCI subunits of the proteasome and the CSN, among them rice Csn8 (p = 0.01) and the drosophila Rpn12 homologue (p = 0.05). All of the twenty top-scoring sequences could be identified as either Csn8- or Rpn12-homologs. As shown in table 1, Csn8 and Rpn12 are the corresponding PCI subunits in the CSN and the lid, respectively. Csn8 and Rpn1 are the most divergent PCI subunits of the proteasome and the signalosome, respectively, and their PCI domains appear to be shorter than that of the more typical family members. Our observations provide good bioinformatical evidence that eIF3k is the fifth PCI-containing subunit of the eIF3 complex and most likely a direct analogue of Csn8 and Rpn12 (figure 2, table 1).

### A structural model for the canonical PCI domain

The discovery of a PCI domain in eIF3k is of particular importance, as a three-dimensional structure of eIF3 has been solved recently [26]. So far, no structural information on the PCI domain has been available, and a structural model for the canonical PCI domain based on the alignment shown in figure 2 should allow interesting insights into the architecture of the PCI complexes.

**Figure 3:**
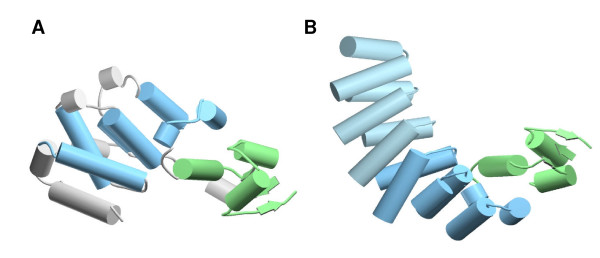
(A) shows the overall structure of eIF3k from the PDB-entry 1RZ4 [26] with β-strands and α-helices represented as ribbons and cylinders, respectively. Regions of the structure with sequence similarity to canonical PCI domain are rendered in colour. Regions belonging to the WH subdomain are shown in green, while conserved structure elements of the helical hairpin regions are shown in dark blue. The connection between β-strand 2 and 3 is not resolved and thus missing in 1RZ4. Other regions (extreme N- and C- termini, connecting helices between hairpins, unstructured regions) are shown in grey. (B) Model of a PCI protein with three additional helical hairpins upstream of the PCI domain. Within the PCI domains, only regions that can be modelled on the eIF3k template are shown. The N-terminal extension is shown in light blue, the other colours are as in figure 3a.

A detailed analysis of the eIF3k structure [26] reveals a bipartite structure of two subdomains that are in close contact through a large inter-domain surface patch (figure 3a). The C-terminal half-domain is a globular α/β structure with an "αβααββ" arrangement. The three β-strands are very short and form an antiparallel sheet. The whole C-terminal part can be classified as a "winged helix" fold and thus is referred to as "WH-domain" [26]. By contrast, the N-terminal half-domain is entirely helical with a core of six regularly-spaced helices that form three antiparallel helical hairpin elements. The resulting superhelix is reminiscent of the solenoids found in helical repeats such as HEAT, Armadillo and TPR. Somewhat unusual are the short 3–10 helices that connect the consecutive α-hairpins. According to Wei et al. [26] the N-terminal half-domain resembles structurally mainly HEAT and Armadillo repeats, and thus the name "HAM-domain" was proposed. The bipartite structure of eIF3k is in good overall agreement with the secondary structure predictions for the single PCI domain families and also with our result of TPR-like helical repeats partially overlapping the PCI domain. It was therefore of special interest to make a detailed comparison of the eIF3k structure and the profile-guided alignment of the canonical PCI superfamily shown in figure 2.

Within the N-terminal subdomain, the sequence conservation between the different PCI domain families is relatively poor and some aspects of the alignment shown in figure 2 are not very reliable. Nevertheless, there is a good correspondence between the helices that build the α-hairpins of eIF3k and the uninterrupted sequence blocks in the PCI alignment. The gap-regions in the PCI alignment are typically caused by insertion events in selected PCI subfamilies. In no case, a deletion of one or more of the hairpin helices is observed. This finding suggests that the helical hairpin structure is conserved in most or all PCI domains. Our alignment and the derived secondary structure predictions suggest that the short 3_10 _helices that connect the helical hairpins in eIF3k are absent in most other PCI proteins. As mentioned in the previous paragraphs, there are several instances of subsignificant sequence similarity to TPR repeats found also *within *the N-terminal subdomain of the PCI domain. By contrast, no similarity to HEAT or Armadillo-repeats has been observed. Thus, we prefer to interpret the helical hairpin structure of the N-terminal subdomain as atypical TPR-like repeats rather than as the HEAT/Armadillo repeats suggested by Wei et al. [26].

The globular C-terminal subdomain (WH) is generally better conserved than the helical N-terminal domain and as a consequence, the part of the alignment covering this structural subdomain shown in figure 2 is more reliable. The "αβααββ" arrangement of α- and β-regions is distributed over two large sequence blocks with a single major gap region between "αβα" and "αββ". As can be seen in figure 2, no important secondary structure element is interrupted by a gap found in the PCI alignment. Like in the N-terminal subdomain, the WH portion shows a good concordance between the secondary structure predicted from the canonical PCI families and the structural elements of the eIF3k structure, apart from minor problems in predicting one of the very short β-strands.

Taken together, the comparison of the PCI alignment with the eIF3k structure (figure 3) shows that the two structures are clearly compatible and suggests that the canonical PCI domains will have an analogous bipartite fold similar to that shown in figure 3. The prediction of TPR-like helical repeats N-terminal of the proper PCI domain suggests that they form an extension of the helical repeat region of the first PCI subdomain. The implications of this model for the overall PCI structure will be discussed below.

## Discussion

### Revision of the eIF3 complex stoichiometry

While the proteasome lid and the CSN complex have an analogous architecture of six PCI-subunits and two MPN-subunits, the more distantly related eIF3 complex has so far only three readily detectable PCI proteins: eIF3a (EIF3S10), eIF3c (EIF3S8) and eIF3e (EIF3S6) [2]. Recent work by Morris-Desbois et al. [34] has grouped eIF3l (EIFS6IP) with the PCI components of eIF3, and our work described above adds eIF3k (EIF3S11) to the ranks of PCI proteins. Besides the PCI subunits, vertebrate eIF3 complexes also contain two MPN proteins: eIF3f (EIF3S1) and eIF3h (EIF3S3). Unlike the situation in the lid and CSN complexes, both MPN subunits of eIF3 have lost their metal-coordinating residues and are most likely catalytically inactive. In addition, several unicellular eukaryotes, including budding yeasts, do not seem to have any eIF3-associated MPN proteins.

Comparing the stoichiometry of eIF3 with the two better-conserved PCI complexes, only one PCI subunit seems to be missing. Our sequence analysis efforts have also included other known eIF3 subunits, but no indications for further PCI domains could be obtained (data not shown). Given the high degree of PCI sequence divergence, it cannot be fully excluded that one of the non-PCI/non-MPN subunits (eIF3b, eIF3d, eIF3g, eIF3i, eIF3j) harbours a cryptic PCI domain that has eluded our detection. On the other hand, it is well conceivable that eIF3 has a deviating subunit composition. In yeast and several other organisms, not only the MPN proteins are missing but also the number of PCI components is reduced, as eIF3e and eIF3l are absent. At present, it is not clear whether the corresponding positions in the complex are left empty or are filled by additional copies of the remaining PCI components. In evolutionary terms, it appears likely that the eIF3 complex is a 'degraded' copy of an ancient lid-like complex, which has lost its MPN+/JAMM mediated catalytic activity and potentially some of its PCI subunits. In turn, by acquiring a group of novel non-PCI/non-MPN subunits, the eIF3 complex has gained a functionality that is different (and potentially even completely unrelated) to the proteasome lid and its cousin, the CSN complex.

### The bipartite structure of the PCI domain

In our original discovery note [2], we had defined the PCI domain as a homology domain, i.e. as a region of localized similarity found within multiple proteins that are otherwise unrelated. The results presented here suggest that this view should be revised. The sequence regions detected as PCI domains by bioinformatical methods seems to consist of two structurally distinct domains. The C-terminal portion, which in eIF3k is referred to as the WH-domain, is much better conserved in sequence than the N-terminal portion, and the C-terminal boundary of the PCI homology domain is relatively well defined by a notable loss of sequence conservation. By contrast, the N-terminal boundary of the homology domain has always been ill-defined, as the overall sequence conservation in this region is low and different families of PCI proteins appear to lose their similarity at different positions. As a consequence, different domain databases and their associated web-servers detect PCI-domains (or the synonymous 'PINT' domains) of varying length in the order PROSITE > Pfam > SMART.

Using the eIF3k-derived structural model, most of these observations can be readily explained. The C-terminal PCI/PINT boundary, which is agreed on by all domain databases, corresponds to the C-terminal boundary of the structural WH-like domain. The N-terminal boundary of the PINT domain, as described in the SMART database, essentially corresponds to the N-terminus of the WH-like domain. The PCI domain of the Pfam database corresponds to the WH-portion plus a single α-helical hairpin repeat. Finally, the PCI domain as described in the PROSITE database covers the WH-portion and all three helical hairpin repeats found in the eIF3k structure. Of the three representations, the PINT domain of the SMART database is structurally most correct, as it describes a true autonomously folding domain. The observation that some PCI families lose their sequence conservation at different N-terminal positions can be explained by assuming a variable number of helical repeat motifs for those proteins. As an extreme example, only the WH-like region could be detected in eIF3e by our profile searches, and the secondary structure prediction for the eIF3e family suggests a β-structure instead of the usual helical-hairpin repeats upstream of the WH region. This finding can be taken as a further hint for structural and functional independence of the N- and C-terminal sub-regions of the PCI homology domain.

### The nature of the N-terminal helical repeat extension

Our finding of TPR-like repeats preceding many PCI domains, combined with the helical repeat structure of the N-terminal portion of the PCI domain itself, leads to the interesting question if these repeats are of the same type and may form a continuous solenoid structure. The authors of the eIF3k crystal structure propose a structural relationship between the eIF3k N-terminus and the HEAT motif based on superposition calculations with DALI [35]. By contrast, our sequence-based analysis methods rather point to an evolutionary relationship to the TPR motif, both for the region preceding the PCI domain and for the first helical hairpin of the PCI domain itself. A related finding was reported for Rpn3 and Csn12 elsewhere, where a homology domain termed "PAM" (PCI-associated module) with TPR-like properties has been proposed [36]. Our findings suggest that the PAM-domain is a special case of a more widespread preference of the WH-portion of the PCI domain to be preceded by TPR-like repeats. In addition, both our results and those of Ciccarelli et al. argue in favour of a continuity between the N-terminal repeats and those found within the PCI domain.

Due to the borderline sequence similarity between the classical TPR motif and the distinct helical hairpins of the PCI proteins, a completely novel type of bi-helical repeats distinct from TPR and HEAT/Armadillo or some kind of intermediary form cannot be ruled out. Structurally, HEAT and TPR repeats are relatively similar and both tend to form superhelical solenoid structures [37]. Without assuming a particular repeat family, we have attempted a rough estimation of what a typical PCI component of the lid or the CSN complex might look like. Figure 3b shows schematically a PCI protein with a WH-like domain at the C-terminus (green), preceded by three helical-repeats assumed to lie within the PCI boundaries according to PROSITE (dark blue), which are in turn preceded by three additional helical repeats that represent the TPR-related N-terminal extension (light blue). As we do not assume a particular repeat family with a well-known radius of solenoid curvature, we use the values derived from the first two helical hairpins of the eIF3 structure instead. It should be stressed that the model of figure 3b with its 'boomerang'-shaped architecture can only give a very coarse approximation of the real situation. Both the solenoid curvature and the exact number of N-terminal repeat extensions are rough estimations. Nevertheless, the model appears to be roughly compatible with the electron density maps of the lid and CSN complexes [4].

### A structural scaffold for three multi-protein complexes

PCI proteins constitute the main components of the proteasome lid and the CSN complex and also form the structural core of the translation initiation factor eIF3. So far, no catalytic activity has been described for PCI proteins. Given the lack of invariant polar residues, such a role appears unlikely. The role of the PCI domains is most likely that of a scaffold for the other complex subunits and other binding partners. There are at least three distinct structural roles that PCI proteins have to fulfil: i) maintaining the integrity of the complex by binding to other PCI proteins, ii) attaching the MPN-subunits to the complex, and iii) binding to other partners such as the base-complex in the case of the proteasome lid or the RNA-binding subunits of the eIF3 complex.

The assignment of these functionalities to the different regions of the PCI proteins, and equally important, the source for the subunit interaction specificity or promiscuity have been subject to several experimental studies, some of them published while others have been presented at a recent meeting on PCI complexes [38]. The PCI model presented here will be certainly useful, both for the interpretation of the experimental results, and for the design of new experiments e.g. those based on domain truncations or domain swaps. According to our analysis, in some proteins the PCI domain is restricted to a C-terminal WH-like part. As these proteins are also components of PCI-complexes, a role of the WH domain in PCI:PCI domain interaction is very likely. On the other hand, TPR-repeats in general form versatile protein-interaction surfaces [29] and we expect the same to be true for the TPR-like repeats found in the PCI proteins.

Tsuge et al. analysed truncated forms of human Csn1 for its interaction with other PCI subunits of the CSN complex [16]. A construct containing residues 197–500 (corresponding to the entire PCI region and some C-terminal material) was able to bind to Csn2, Csn3 and Csn4. Another construct starting at position 340, and thus lacking the helical-repeat region, no longer bound to Csn2 and Csn4 but maintained binding to Csn3. By contrast, a construct 197–307 that lacks the WH-like region was only able to bind to Csn4. These experiments suggest that both the WH portion and the helical-repeat part of the PCI proteins have a role in PCI:PCI interactions, although they seem to interact with different subunits of the complex. The importance of both subdomains is confirmed by a recent study of Isono et al., who analyse multiple point mutations in the lid subunit Rpn7 [39]. In their hands, both mutations in the N-terminal helical repeat part of Rpn7 and mutations within the WH-like region are able to abrogate binding to Rpn3, another PCI subunit of the proteasome lid. So far, no information is available on the PCI regions involved in binding to the MPN subunits.

## Conclusion

In summary, we believe the PCI domain could play a role as a universal binding domain supporting intra-complex interactions as well as recruitment of additional ligands. The model presented here is a first step to the understanding of the supramolecular architecture of three important complexes and certainly will facilitate the interpretation of further experimental results. Nevertheless, a full understanding of the interaction mode between PCI- and MPN-domain proteins will certainly require experimentally determined high-resolution structures of the components – or ideally, that of an intact complex.

## Methods

### Database searches

Sequence database searches were performed with a nonredundant data set constructed from current releases of SwissProt, TrEMBL, and GenPept [40,41]. Generalized profile construction [20] and searches were run locally using the *pftools *package, version 2.1. (program available from the URL ). Generalized profiles were constructed using the BLOSUM45 substitution matrix [42] and default penalties of 2.1 for gap opening and 0.2 for gap extension. Statistical significance of profile matches was derived from the analysis of the score distribution of a randomized database [43]. Database randomization was performed by individually inverting each protein sequence, using SwissProt 34 as the data source. Only sequence matches found with a probability of p < 0.01 were included into subsequent rounds of iterative profile refinement.

### Multiple alignments

For sufficiently related proteins, multiple alignments were calculated by T-coffee [44], using excised domains instead of the entire sequences. For alignments of highly divergent sequences, such as the whole PCI family, the overall alignment was generated by profile-guided assembly of family-specific subalignments. If necessary, manual adjustments were introduced in the final alignment step. Whenever possible, positions for insertion- and deletion events were placed according to predicted secondary structure elements derived from the subfamilies involved.

### Structure prediction

For each subfamily-specific multiple alignment, secondary structure elements were predicted using web services of PHD [30] and JPred [31]. JPred uses a set of different algorithms for secondary structure prediction and calculates a consensus prediction. By combining the JPred and PHD derived secondary structure predictions, every position within a given alignment was assigned to "helical", "sheet" or "none". For attempting a sequence-based fold recognition, representative sequences were submitted to the 'Superfamily' web service [32].

## Authors' contributions

HS performed the sequence analysis and participated in writing the manuscript. KH designed the study, participated in the sequence analysis and in writing the manuscript. All authors read and approved the final manuscript.

## Figures and Tables

**Table 1:** PCI complexes and their subunit correspondence

**Domain**	**proteasome lid**	**CSN**	**eIF3**
PCI	Rpn7 / PSMD6	Csn1	eIF3a, eIF3c, eIF3e, eIF3l
PCI	Rpn6 / PSMD11	Csn2	eIF3a, eIF3c, eIF3e, eIF3l
PCI	Rpn3 / PSMD3	Csn3	eIF3a, eIF3c, eIF3e, eIF3l
PCI	Rpn5 / PSMD12	Csn4	eIF3a, eIF3c, eIF3e, eIF3l
PCI	Rpn9 / PSMD13	Csn7a,b	eIF3a, eIF3c, eIF3e, eIF3l

PCI	Rpn12 / PSMD8	Csn8	eIF3k
MPN+	Rpn11 / PSMD14	Csn5	-

MPN	Rpn8 / PSMD7	Csn6	eIF3f, eIF3h
